# Evolution and challenges of sport policy in Lithuania: a historical and contemporary analysis

**DOI:** 10.3389/fspor.2025.1659099

**Published:** 2025-10-17

**Authors:** Rimantas Mikalauskas, Irena Valantine, Marko Begović

**Affiliations:** ^1^ Department of Sport and Tourism Management, Lithuanian Sports University, Kaunas, Lithuania; ^2^ Faculty of Business Administration and Social Sciences, Molde University College, Molde, Norway

**Keywords:** sport policy, Lithuania, stakeholder involvement, Soviet system, democratic model, governance

## Abstract

**Introduction:**

This paper examines the evolution and contemporary challenges of sport policy in Lithuania, emphasizing the interplay between politics and national planning. It provides a historical overview of Lithuanian sport policy development, particularly focusing on the transition from the Soviet system to a democratic model after regaining independence in 1990. Key components, stakeholders, and the structure of the current Lithuanian sport system are analyzed, highlighting the critical role of sports science. The study identifies significant gaps in academic research on sport policy and underscores the necessity for more comprehensive studies in this field. It discusses the main challenges influencing the future of Lithuanian sport policy, including political instability, bureaucratic influence, and limited stakeholder involvement. The purpose of this paper is to address a gap in the academic literature and enhance the scientific understanding of sport policy development in Lithuania. Accordingly, this study seeks to answer the following research question: How has Lithuanian sport policy evolved since independence, and what political and institutional factors currently shape its development and implementation? By exploring this question, the study aims to fill a notable gap in academic literature and contribute to a deeper understanding of sport governance in post-socialist European contexts.

**Methods:**

The study applies a qualitative document based analysis covering Lithuanian sport policy from 1990-2025.

**Results:**

The findings reveal that Lithuanian sport policy remains highly centralized and politically influenced, with fragmented governance and limited stakeholder involvement.

**Discussion:**

The study concludes with a call for a unified long-term national strategy, transparent funding mechanisms, and evidence-based policymaking to strengthen future sport governance.

## Introduction

Politics plays a key role in national planning and decision-making. Quality implementation of national sport policy is one of the key factors for the country's prosperity and competitiveness in global processes. This paper offers an overview of the evolution of sport policy in Lithuania, including its sports structure, key stakeholders, essential components, and the challenges that will shape the future development of sport policy in the country. A detailed examination of this evolution and the institutional capacity for change within the Lithuanian sport system is also offered by Čingienė and Mizeras (1), who highlight the influence of political, administrative, and cultural transitions on governance structures.

Research on sport policy in Lithuania has never been a priority for the academic community, but some attempts have been made to review the existing sport system (2–6). Only one component of sport policy has been analysed in sufficient detail and actively—sports science, which is the main interest of academics (7–9). Lithuanian sports scientists have essentially fulfilled two of the four functions of science: providing scientific services and fostering a science-sensitive community. On the other hand, Lithuanian sports scientists fulfilled an important mission: by using Lithuania's scientific potential, they helped coaches to manage the training of athletes in a targeted way to achieve the best personal performance at the Olympic Games; they provided the latest knowledge in sports science, improved the qualifications of coaches, and fostered a science-intensive sports community (9). According to the political and policy perspectives outlined by Houlihan, B., White, A. (10), and Chaker, A-N. (11), the current approach to studying sport policy includes analyzing legislation, policy documents, and government and other reports. The effectiveness of sport policy implementation and the success of the national sport policy model are contingent on the successful transformation of the country's national sport institutions. Additionally, a historical overview is crucial for understanding the development of sport policy and its influence on the evolution of the sport system.

After World War II, Lithuania's physical education system was managed under Soviet structures. In 1953, the Committee for Physical Education and Sport was replaced by the Chief Board for Physical Education and Sport within the LSSR Ministry of Health, integrating local committees into health departments. This system proved ineffective, and in 1954 the Committee of Physical Education and Sport under the Council of Ministers was restored, with district-level sports led by full-time staff.

Following Lithuania's 1990 restoration of independence, the Physical Education and Sports Committee and Lithuanian National Olympic Committee (LNOC) urged athletes to boycott USSR competitions. In April 1990, the Department of Physical Culture and Sports (KKSD) replaced the Soviet-era committee, marking the start of a transition to a global sports governance model. A Western-style club system was established, federations gained independence, and new public sports organizations such as the Paralympic Committee and the Union of Lithuanian Sports Federations were formed.

By 1996, the 2nd Lithuanian Sports Congress recognized the national sports model as aligned with Western democratic standards and increasingly integrated with international programs. This historical overview sets the stage for this paper's focus on key actors and components of the modern Lithuanian sport system. It aims to fill a gap in academic literature and explore the primary challenges shaping future research on sport policy development in Lithuania.

This study addresses this gap by examining the evolution and challenges of Lithuanian sport policy since independence. Specifically, it seeks to answer the following research questions: (1) How has Lithuanian sport policy evolved since 1990? (2) What political and institutional factors have shaped its development and implementation? By situating Lithuania's case within broader debates on post-socialist sport governance, the study contributes both an empirical account and theoretical insights into the role of politics, institutions, and international influences in shaping national sport systems.

## Materials and methods

This study employs a qualitative document analysis approach. The analysis covers the period from the restoration of Lithuanian independence in 1990 to the most recent legal and policy developments in 2025. Primary sources include national legislation (e.g., the Law on Physical Education and Sport), strategic policy documents, parliamentary resolutions, reports by the Ministry of Education, Science and Sport, and publications of the National Sports Agency. Secondary sources include academic studies and international policy reports. Documents were selected for their relevance to sport governance, legal significance, or impact on institutional structures. While this approach enables a comprehensive overview of policy evolution, it is limited by its reliance on documentary sources, without supplementary interviews or survey data. Future research could complement this study with stakeholder perspectives to provide a deeper understanding of policy-making dynamics.

The approach integrates historical analysis and policy analysis to trace the transition of Lithuanian sport governance from the Soviet era to the present democratic model. The research is grounded in the examination of official documents, legal texts, policy reports, and academic literature relevant to sport policy in Lithuania. Key sources include national legislation (e.g., the Law on Sport), strategic policy documents, reports from governmental bodies such as the Ministry of Education, Science and Sport, and the National Sports Agency, as well as publications by Lithuanian and international scholars. The analysis covers documents produced between the post-World War II Soviet administration of sport in Lithuania, the 1990 restoration of independence, and subsequent developments through the most recent legal and policy updates in 2025. By employing this methodology, the study aims to identify key institutional actors, map the structural development of sport governance, and analyze the political, legal, and administrative barriers that continue to shape sport policy in the country. This qualitative approach allows for a comprehensive understanding of the interplay between politics, public administration, and the sport sector in Lithuania.

## A historical overview of the evolution of Lithuanian sports policy

The former socialist system sought to use physical culture and sport as a means of mobilising society to address political, cultural and economic challenges. In Lithuania, the aim was to closely interlink the two strands of the physical culture and sports movement: mass and mastery. Unfortunately, in terms of mass participation, this was done only formally. The fact that sport and its achievements were elevated to the level of state policy was skilfully used by Lithuanian sports specialists to create a distinctive system of training athletes that was more in line with our national traditions (12).

The Lithuanian National Olympic Committee (LNOC), which was re-established on 11 December 1988, gave a major impetus to Lithuanian sports policy. The LNOC sought to accelerate the reorganisation of Lithuanian sport, and began to take care of the problems of big sport. Lithuanian sports organisations, their leaders and other specialists, while formally pursuing the official policy, were also able to pursue a real and distinctive sport policy that served the interests of Lithuania (13).

The role of the state in shaping and developing sport policy in the country should also be emphasized. Two stages are distinguished: before independence and after Lithuania became independent. In the first stage (1940–1990), after the Soviet Union occupied Lithuania, the development of sport policy was ideologized and reorganized according to the USSR model (14). In 1990, after Lithuania regained its independence, the state became the main shaper of sport policy in the country. The main institution was the Department of Physical Culture and Sports under the Government of the Republic of Lithuania. Since 2019, after the Law on Sports came into force, the Ministry of Education, Science and Sports has been tasked with shaping and implementing the state sports policy. In addition, the development of sport policy has relied on two interconnected normative sources: (1) the sport-specific regulations of the International Sports Federation (ISF) and (2) the rules of the National Sports Federations (NSFs), which are rooted in the country's constitutional framework and its political and legal system. The policy framework was established based on the internal regulations of the sports movement, relevant laws (including by-laws), intergovernmental agreements related to sport, government contracts in the field of sport, and established norms and rules governing sports activities. The 1st Lithuanian Sports Congress in 1993 discussed and endorsed the basic principles of the Law on Physical Education and Sport, adopted the Sports Charter, and outlined the goals and objectives of the independent Lithuanian sports movement. Decision-making became more democratic, and cooperation between sports organisations improved (12).

In 1991, the Sport for All Association was founded, which successfully organised the traditional Sport for All festivals and other mass events. In 1994, the Women's Sports Association was founded and women's sports games and festivals began to be organised. Attention was paid to people with disabilities. In 1991, the Paralympic Committee was established, and other sports organisations such as Blind Sports Federation, Deaf Sports Committee, Lithuanian Disabled Sports Federation, Special Olympic Committee were created (13).

The experience of democracies around the world has encouraged the development of the legal foundations of a national sport system. Article 53 of the Constitution of the Republic of Lithuania proclaimed that “The state promotes physical culture in society and supports sports” (15). The participants in physical culture and sport included athletes, professional sportspeople, physical culture and sports clubs, municipalities or their authorized bodies, regional or national sports federations (by branch), athlete education centers, county governors, departments of county governors' administrations overseeing sports, state institutions and regulatory bodies involved in the development of physical education and sport, schools and sports centers promoting healthy lifestyles, and other organizations focused on advancing physical education and sport.

Sports organizations (sports clubs) encompassed legal entities such as sports clubs, sports schools, sports centers, sports facilities, sports federations, associations, societies, and other institutions involved in physical culture and sporting activities. These organizations facilitated the practice of physical culture and sport, the training of athletes, and the organization of sports competitions and other related events, thereby shaping the core elements of sports policy.

The procedure for the establishment of sports organisations and the accreditation of their activities were established in documented legal acts—the Law on Public Enterprises of the Republic of Lithuania (16), the Law on Associations of the Republic of Lithuania (17).

In 1998, the following organisations in the public sector were involved in physical education and sport in the Republic of Lithuania, as well as other organisations involved in the development of sport: Commission for Youth and Sports of the Republic of Lithuania, the Department of Physical Education and Sports (KKSD) under the Government of the Republic of Lithuania, the Ministries of National Defence and the Ministry of the Interior of the Republic of Lithuania, the Ministry of Education and Science of the Republic of Lithuania, the Higher Education Institutions of the Republic of Lithuania, the Lithuanian Centre of Schoolchildren's Physical Education and Sports, the County Sports Councils, District and City Public Health Offices, the Municipal Physical Education and Sports Departments, Municipal sports schools, Physical education and sports centres and their sports facilities. As noted by Čingienė (18), the organisation of sport governance in Lithuania has historically reflected a top-down model, with strong public sector involvement and hierarchical decision-making rooted in earlier political systems.

To improve coordination in physical culture and sport, Lithuania's government was advised to create mechanisms aligning institutions responsible for sports policy. This aimed to ensure consistent development nationwide and boost youth participation. By 2002, the number of organizations promoting sport had grown significantly. New public sector entities included the President's Adviser, Ministries of Social Security and Labour, Foreign Affairs, the Expert Commission on Strategic Issues in Lithuanian Sport, the Working Group on High-Performance Athlete Training, and the Lithuanian Sports Information Centre. Private companies like UAB Sports Testing and Rehabilitation Centre also emerged to support the sector.

However, since the restoration of independence in 1990 and until 2009, a provision of the Law on Physical Education and Sport has remained unimplemented, namely the lack of a national strategy for physical education and sport. Only two strategies (State Strategy for the Development of Physical Culture and Sport 2009–2020) (19) and The National Strategy for the Development of Sport for All (National Strategy for the Promotion of Physical Activity of the Lithuanian Population 2008–2020) related to the development of physical culture and sport were created and submitted to the Seimas of the Republic of Lithuania for consideration, but none of them was adopted. The failure to adopt these strategies indicates that sport policy has not been seen as a long-term political priority in Lithuania. There could be a range of reasons why this outcome occurred. First, shifting political agendas and frequent government reshufflings meant that sport was always in the shadow of policy fields such as health, education, or economic reform. Second, legislative support for sport policy was irregular, a reflection of the perception that sport is a secondary matter and not a part of mainstream national construction. Third, institutional weaknesses—including intra-ministerial conflicting mandates and poor stakeholder engagement—most likely undermined the strategies' legitimacy. Finally, content-based limitations, such as the absence of clear implementation mechanisms, funding structures, and measurable indicators, further reduced their uptake potential. As they interacted, they indicate the manner in which political instability, sporting low priority, and technical deficits in strategy formulation operated to prevent the formulation of a comprehensive national sport policy framework.

In this context, sports policy, sport itself, has not been recognised as a key priority of the state, cooperation between the education, social security, and health care systems has not been systematically promoted, only short-term programmes and plans for the development of sport have been implemented, and they have not included targeted and consistent coordination of the actions of the state—the President, the Parliament, the Government (ministries), municipalities, other institutions, businesses, research and study institutions, and NGOs—and their efforts have not been mobilised in order to bring about fundamental changes in the area of sports policy.

## Sport in the modern public sector

Since Lithuania's accession to the European Union in 2004, the Lithuanian sports market has increasingly integrated into the broader European sports structure, where globalisation, competition and commercialisation have gained particular importance, which, according to Houlihan and Green (20), has significantly worsened the perception of amateurism in sport. Article 53 of the Constitution of the Republic of Lithuania (15) mandates that “The state promotes physical culture in society and supports sports”. Due to shifting political priorities, the Seimas of the Republic of Lithuania has frequently altered the institutional environment for sport, and the government has placed control over sport under the Department of Physical Culture and Sports (KKSD).

The legal framework for physical culture and sports organizations, the roles of state institutions in this field, the regulation of professional sports, legal assurances for the public to engage in physical culture and sports, and the regulation of other related matters were established. The Law on Physical Culture and Sport of the Republic of Lithuania was drafted in 1995 and later that year adopted by the Parliament, which became the basis for legal regulation of sport (21). Specifically, the lawoutlined that the physical education and sports system comprised several key areas: educating children and youth in physical education and sports, promoting physical education for adults and people with disabilities, advancing sports development, and training elite athletes.

In 2009, on the proposal of the Commission for the Improvement of State Management (“Sunset” Commission), the Government of the Republic of Lithuania decided to change the status of the Department from a governmental body to a body attached to the Ministry of the Interior, and to delegate the functions of participation in the policy-making process in this area (drafting of legal acts) to the Ministry of the Interior (22). The KKSD in 2012 organised its activities in accordance with the Law of the Republic of Lithuania on Physical Culture and Sport, the Law of the Republic of Lithuania on the Fund for Support of Physical Culture and Sport, and the State Strategy for Sports Development 2011–2020.

The Seimas adopted a resolution on sport policy (23) emphasising that it would implement the National Sports Development Strategy 2011–2020 (24), the Lithuanian Health Programme 2014–2025 (25), which establishes long-term commitments to creating a healthier society, establishes goals and objectives for health promotion activities, and aims for health indicators and its guiding principle “health in all policies”, the National Education Strategy 2013–2022 (26) “On the approval of the National Education Strategy 2013–2022” and the European Strategy for Child and Adolescent Health 2015–2020 (the indicators and objectives for evaluating the implementation of this strategy emphasize the efforts of the education community for fundamental changes in education, turning Lithuanian education into a sustainable basis for improving the welfare of the state). However, a monitoring system for the implementation of the strategy (National Sports Development Strategy 2011–2020) was not created, and there was no internal control system in place, based on reliable indicators clearly defined in the Strategy, especially in the implementation of the 2011–2020 State Sports Development Strategy and its compliance with state policy priorities. The main indicator set out in the National Education Strategy 2013–2022 is that by 2025 the general average life expectancy limit would be 77.5 years. The program establishes 4 goals: to create a safer social environment, reduce health inequalities and social exclusion, create a health-friendly working and living environment, form a healthy lifestyle and its culture, ensure high-quality and effective health care focused on the needs of the population.

The aim of the Health Saving and Promotion Policy Guidelines (2019) was to initiate a new policy for saving and promoting health in Lithuania, to set out the key policy directions for saving and promoting health in Lithuania, and to set out the strategic objectives that were necessary to achieve the goals set out in the State Progress Strategy “Lithuania 2030” (27).

The Parliamentary Resolution on the Adoption of the Policy Guidelines on Health Saving and Promotion (28) provided for measures and called for the Health Saving and Promotion Policy Guidelines should be considered when preparing the Health Saving and Promotion Policy Guidelines for 2019. The draft law for approving the financial indicators of the state and municipal budgets is anticipated to be adopted by the Council of Ministers in 2018. But in 2019, a resolution was adopted on the liquidation of the Department of Physical Culture and Sports under the Government of the Republic of Lithuania (28). With the abolition of the KKSD, the responsibility for sport policy in the country was transferred to the Ministry of Education and Science, with the addition of the name Ministry of Education, Science and Sport. Three years later, the National Sports Agency was established (Government Decision No 418 “On the Establishment of the Budgetary Institution of the National Sports Agency”), with the aim of implementing the State's sport policy in the fields of highperformance sport and physical activity. The Agency also concentrated the sport financing functions of the whole country, with equal emphasis on Olympic and Paralympic sport and public physical activity. The Agency is charged with looking after sports infrastructure and athletes' rights, and with assisting the country's sports federations and organisations in introducing and strengthening the principles of good governance.

According to Chaker (11), Lithuania's sport system is interventionist, with regulated structures, development processes, and defined roles for public and non-profit entities. The government played a key role in creating the Law on Physical Culture and Sport (1995), which establishes sport as a public interest activity, sets the organizational framework, and defines funding mechanisms. Lithuania's sports system is also governed by several international laws and conventions, including the European Conventions on spectator safety and the Council of Europe's integrated approach to football match security (29), as well as the International Convention against Doping in Sport (30). These international treaties become part of national law after ratification by the Lithuanian government and Parliament. Lithuania joined the Anti-Doping Convention in 1996, following ratification by the government in 1989, aiming to combat doping in sport. In 2006, Lithuania became a party to the UNESCO International Convention against Doping in Sport, committing to implement its provisions through constitutional resolutions and national legislation.

Lithuania has a “combined sports funding model”, involving public funds (state and local budgets), companies and private individuals. The National Sports Agency (NSA) administers the awarding of scholarships, prizes and rents to state athletes, launches competitions for physical activity projects and programmes, and administers funds for high-performance sport, physical activity and sports infrastructure. The annual budget is around €60 million, of which State funding for high-performance sport has increased by more than 60% in 2021, with a total of €15.9 million, €22.7 million in 2022 and almost €30 million in 2023. Federations have the responsibility to develop their sport, to represent the interests of their members, to set and/or enforce requirements specific to their sport, and to develop and implement various measures to promote their sport (31). The draft Government Programme Implementation Plan also includes more effective structures for sports management and sporting achievements: to develop sports infrastructure on the basis of criteria and data from the sports register; to specify the assessment criteria for the activities of the federations and the performance model of the Sport Support Foundation; to set up a centre of excellence to provide methodological support to federations in implementing the principles of good governance; to prepare and implement a plan for the development of sports gymnasiums and sports classes. Measures for the development of sports infrastructure and improvement of training of high-performance athletes are also provided for in the draft Sport Development Programme prepared for the implementation of the National Progress Strategy (National Audit Office of Lithuania, 2021). However, the Ministry of Education, Science and Sport has provided information on planned major changes to the Law on Physical Education and Sport: clarification of the definition of high-performance sport, the establishment of a register of sports, the separation of the funding of physical activity and high-performance sport, an increase in state grants for athletes, the addition of a requirement for federations to have their funding criteria audited, the submission of sports data and financial statements to registers, and the establishment of sports performance contracts with athletes. The criteria for strategic sports are planned to be linked not only to achievements, but also to the popularity and spread of the sport, and the results of the organisation of federations (32).

A second source of funding to promote the development of physical activity in the country is national physical activity programmes. Adopted in 1995, the law establishes the principles of sport, the sport system and its governance, regulates the competence of state and municipal authorities and bodies in the field of sport, the activities of the athletes' representative, the requirements for coaches and instructors of physical activity or high performance sport, the organisation of sport, the health check of persons, the security requirements for sport events, the financing of sport, the implementation of the policy of anti-doping and the fight against manipulation of high performance sport competitions, and the state promotion of achievements in high performance sport (33).

The Council of Europe Convention on the Manipulation of Sporting Events (the Macolin Convention) (34), which focuses on international cooperation and prevention measures, risk assessment and management, information exchange, protection of personal data, law enforcement cooperation, and the establishment of liability (criminal and administrative), was signed in 2014 and has 39 signatories. Once ratified by States, it is expected to become an effective tool in the fight against this phenomenon at international level. Under the Law on Sport, a national physical activity program is a four-year planning document created by a national umbrella non-governmental organization. It outlines the objectives, targets, and actions to achieve them, as well as the timelines, evaluation criteria, and consequences in accordance with the guidelines set by the Minister for Education, Science, and Sport. The purpose of these national physical activity programs is to systematically enhance the physical activity levels of the population, and they are distinct from national or regional physical activity projects. The amount of eligible funds requested from the Sport Support Fund for the implementation of a regional physical activity project may not be less than 5 000 EUR (five thousand) and may not exceed 50 000 EUR (fifty thousand). The National Sports Agency has also allocated 20 million EURof the state budget for 2023 for the High Performance Sport Programmes, for the implementation of the four-year program has been allocated almost 1.9 million EUR. The National Physical Activity Programme for the organisation of mass physical activity events is planned to be financed by EUR 450 000 in 2024, EUR 600 000 in 2025, EUR 600 000 in 2026, and EUR 600 000 in 2027. Applicants must contribute at least 1% of the programme's cost estimate from their own or other sources (35).

The third source of public funding is the distribution of lotteries and gambling revenues. Currently, lottery organisers pay 5% to the budget and beneficiaries 8% of the value of the tickets distributed. In 2023, €5.356 million will be transferred to the budget (€5.632 million in 2022) and €8.505 million (€9.111 million) to beneficiaries. The LNOC received €30.572 million in support from lottery organisers between 2015 and 2020, while the Lithuanian Olympic Foundation received €2.226 million. In 2023, the Seimas decides to start the procedure for consideration of amendments to the Law on Lotteries and the Law on Lotteries and Gaming Tax. The amendments to the laws foresee a doubling of the lottery tax rate by changing the tax base from the current 5% rate on turnover to 10% on the balance after payment of winnings. It also seeks to increase the share of the proceeds from ticket sales that must be allocated to charity and aid from 8%–16%. This is expected to deprive the State of almost €2 million a year in budget revenue and will lead to a boom in gambling. After the implementation of the amendments to the law, lottery organisers would have to contribute 16% instead of 8% to the support, and would also pay a base tax of 10% on the funds remaining after the payout of winnings.

## Non-profit sports sector

While the government establishes the framework and guidelines for sports policy, the actual implementation of these measures relies on the sports movement, including public sports organizations like the Lithuanian Association “Sport for All,” the Union of Lithuanian Sports Federations, and the Lithuanian National Olympic Committee. The Lithuanian sports system is characterized by a bureaucratic structure, as evidenced by parameters identified by the VOCASPORT Study Group: the role of public authorities, the level of coordination and involvement, the functions of the voluntary, public, and private sectors, and the system's adaptability (VOCASPORT Study Group 2004).

Established in 1988, the Lithuanian National Olympic Committee (LNOC) is an independent, not-for-profit, non-govermental organisation belonging to the world Olympic Movement. In its activities, the LNOC is guided by the Constitution and laws of the Republic of Lithuania, the Olympic Charter, the anti-doping code of the Olympic Movement, the decisions of the International Olympic Committee (IOC), and the statutes of the LNOC. It promotes the basic principles of Olympism in the country's sporting activities, seeks to reflect Olympism in the physical education and sports programmes of schools and universities, takes care of the creation of Olympic education institutions and promotes the development of sport. LNOC transports athletes to represent Lithuania at the Olympic Games and other Olympic events, together with Lithuanian sports federations, unions, associations and other institutions, takes care of the selection and preparation of athletes for the Olympic Games and other competitions under the auspices of the IOC, participates and sends athletes to the Olympic Games, the European Games, the Youth Olympic Games and Olympic Festivals. Together with the LNOC, the Lithuanian Association “Sport for All”, the Union of Lithuanian Sports Federations, and the Lithuanian Student Sports Association, they can be considered as the main institutional bodies for the development of grassroots sport, which administer and coordinate sport and physical activity activities at the national level and contribute to the promotion of sports. Founded in 1991, the Lithuanian Association “Sport for All” is an NGO uniting on a voluntary basis the republican non-governmental organisations of physical culture, sport and tourism registered with the Ministry of Justice of the Republic of Lithuania, which develop health enhancing physical culture and amateur sports. The primary goals of the Association are to enhance public welfare through physical culture and sport by improving individuals' health, encouraging healthy lifestyles, and organizing physical culture, amateur sports, and recreational events for people of all ages. Lithuanian Student Sports Association (LSSA) is an independent public organisation (founded in 1990). LSSA coordinates university sports activities, promotes physical and spiritual education of academic youth as a philosophy of healthy life, sports values, cooperation with student sports organisations of other countries, distances itself from discrimination, violence, doping.

The Union of Lithuanian Sports Federations, founded in 1992, is an independent, nonprofit, limited civil liability organisation established in accordance with the Law on Associations of the Republic of Lithuania and other legal acts, uniting sports federations, associations and unions registered in the Republic of Lithuania, which are formed on a voluntary basis, to take care of the popularisation, promotion and development of sports in Lithuania, in co-operation with sports and other organisations.

## Contemporary sport policy issues

Lasswell (36) suggested that politics is fundamentally a contest for power and influence, where those who dominate these positions in society are able to make decisions that impact every citizen's life. According to Bergsgard et al. (37), sport's unique nature means it serves as both “a significant public service and, in many countries, a crucial component of general social provision,” as well as “an important economic factor through job creation, capital investment, and workforce balance” (2007:3–4). Three key issues are likely to shape the future of sport policy in Lithuania: (1) structural, regulatory, and legal obstacles, (2) the effects of politicization on sport governance, and (3) the implementation of internationally binding standards. These issues are critical due to their impact on sport policy and the media's scrutiny of various governmental actions (38). That said, the institutional framework presented before signals a number of similarities in identifying and explaining sport ecosystem from policy processes and relationship primarily between public and sport sector. Furthermore, Begović (39) found joint determinant of countries from the Central and Eastern Europe toward transitioning from socialism to capitalism, especially in respect the structure and composition of sport-related institutions. The degree of centralization and interventionism and the concentration of power, suggest that sports sector tend to follow or mimic dominant political structure exercised through “competitive authoritarian” system blends aspects of both democratic and authoritarian governance (40). As Čingienė and Gobikas (41) argue, Lithuanian sport policy is still heavily influenced by hierarchical governance structures, which complicate coordination across institutions and hinder policy flexibility.

## Structural and regulatory barriers

Lithuania is a small country and the strategic orientation of such countries can be found in Houlihan and Zeng's (65) description of the concept of isomorphism/imitation. Drawing from these authors' observations, Begovic (38) contends that states aim to safeguard their own interests by assuming control over the interests of sport. Lithuanian politics is characterised by a high level of state intervention with bureaucratic configurations. To effectively analyze sport policy and the structure of sport, it is essential to have a clear understanding of the state's role, including its structure, the market, and aspects of civil society, despite the fact that these “social orders” are frequently interdependent. The state creates the structures that govern societies themselves. This refers to the state system for the organisation and management of sport, in which, in addition to the ministries and their subordinate bodies, various organisations operate. For example, at municipal level, we have sports schools, physical education and sports centres, boarding schools or sports gymnasiums. While sport schools are established and overseen by local authorities, their functions are largely shaped by centralized public policy goals and regulations defined at the national level. Meanwhile, the market essentially refers to business activity, the position of the private sector. Civil society typically consists of a network of informal, non-market relations based on households and active communities. The market essentially refers to business activity, the situation of the private sector. Civil society consists of a network of informal, non-market relations based on households and active communities. As Hoye and Doherty (66), Hoye et al. (67), Kobayashi et al. (68) argue, the intersection of such three social orders creates four distinct sectors: public, commercial, informal and voluntary.

In 2004, when Lithuania became a member of the European Union, the Government of the Republic of Lithuania prepared a program for the development of physical education and sports. The program emphasized most strongly the social function of sports, as well as the factors of education and development of society through sport. Less emphasis was placed on the importance of sport for strengthening the country's international image, promoting healthy lifestyles and social cohesion, and the significance of major sporting achievements for the state. The most prominent focus of the Government's physical education and sports policy was the improvement of cultural and sports infrastructure.

The Law on Physical Culture and Sports (21), as well as Article 6 of the Law on Local Self-Government, clearly defined the competence of municipalities in the field of physical education. However, municipalities were not financially independent; their ability to form budgets and taxes was limited, they did not control land, and their financial system was unclear. This did not ensure sufficient independence for them, nor proper development and implementation of autonomous functions (Conference “State Sports Policy: Problems and Solutions”, 2007).

On the other hand, Lithuanian society's attitudes and habits did not change at all from 2001–2007. Lithuania remained among those post-Soviet countries that oriented their sports policy and directed most of their resources solely toward the cultivation of achievement (elite) sports, while no institution essentially assumed final responsibility for the physical activity of the population, especially older people.

In 2007, a working group was assembled to prepare a draft national strategy in the field of physical education and sports (Order of the Prime Minister of the Republic of Lithuania, July 13, 2007, No. 259), which was titled the Draft Strategy for the Development of Physical Education and Sports (2008–2020). The authors of this project focused primarily on the social mission of sport, aiming to create conditions for involving all social groups in physical education and sports activities. After discussions, the project was improved and presented as the 2010–2020 National Sports Development Strategy project, which was approved by the Seimas in 2010 (Resolution of the Seimas of the Republic of Lithuania “On the Approval of the Strategy for Promoting Physical Activity of the Lithuanian Population for 2008–2020” (42) project (XP-2797), November 10, 2010, No. 106-P-52, Conclusions of the Seimas Committee on Education, Science and Culture).

Although the strategy was prepared, many provisions and documents outlining how and in what way physical activity promotion in Lithuania should be improved, as well as numerous scientific and commissioned studies on the subject, only revealed the most pressing problems-yet in reality, everything remained only on paper. The end of socialism in Lithuania did not change the level of state intervention; rather, modern sports structures were formed, but without a perfected horizontal public administration system for sports that would encompass many areas of state governance and municipal institutions.

A historical study of the determinants of the Lithuanian sport system suggests that it has not been able to offer sustainable solutions. In other words, the end of socialism in Lithuania has not changed the level of state intervention, but has only led to the emergence of modern sport structures, without a perfect horizontal structure of public administration of sport, encompassing many spheres of state management and municipal institutions, which would allow for the effective development of cooperation with non-governmental sports organisations and the private sector of sport activities.

In order to maintain the status quo, the aim was to create conditions for the involvement of all social groups in sport in Lithuania, thus addressing the crucial challenges of social cohesion, improving the quality of life, the health and healthy lifestyles of the population, increased working capacity, and leisure time employment, while creating a social basis for a system of training high-performance athletes. The main role here was once played by the KKSD, whose main objective was to participate in the formulation of public policy in the field of physical education and sport and to implement this policy. Today, however, the activities of the National Sports Agency (43) show that sport is undeservedly isolated as a separate, small sector of the public administration, whereas it is in fact a global, i.e., inter-departmental, area, which is an important function of municipal authorities, an important part of the country's overall economy, and must therefore be broadly inclusive of NGOs, public organisations and private business initiatives. To explain this situation, we need to look back a little. Firstly, back in 2009, the European Commission had already adopted a new approach in the field of public-private partnerships (IPPs). In 2009, the report of the State Audit Office of the Republic of Lithuania on the development of physical culture and sport, among other shortcomings, highlighted the fact that the Department of Physical Culture and Sport did not ensure proper performance of the functions assigned to it, and did not have an effective monitoring and control system in place for obtaining information from institutions and organisations, The State Audit Office of the Republic of Lithuania did not initiate amendments to legal acts and/or envisage the necessary measures, but only individual programmes and projects were financed, and the objectives of the implemented programmes were often overlapping (State Audit Report of the State Audit Office of the Republic of Lithuania, 2009, No. va-p-50-1-28). Secondly, the establishment of the National Sports Agency (44) has as its main objective the implementation of a national sport policy in the fields of high-performance sport and physical activity. The Agency also takes care of sports infrastructure and athletes' rights, and assists the country's sports federations and organisations in introducing and strengthening the principles of good governance. In this context, the National Agency for Sport does not deal with deep issues of sport policy design and development, but essentially only with the effective management of resources to encourage organisations to create a physically active and healthy society and to create conditions for talented athletes to flourish [2014-2020 National Progress Program (2012) (69), 2021-2024 Government Program (45), 2008–2012 Program of the Government of the Republic of Lithuania (70)].

In 2010, Lithuania's Ministry of the Interior was assigned responsibility for physical culture and sport, emphasizing physical activity as essential for health and social cohesion (Strategic Plan 2011–2013). However, in 2019, the Government abolished the Department of Physical Culture and Sport, despite the European Commission's new strategy promoting physical activity and health-enhancing physical activity (HEPA) across sectors. The Council recommended regular reporting on HEPA implementation, highlighting its importance for health, productivity, and the Europe 2020 goals. Member States were expected to evaluate the added value of these efforts based on a monitoring system. In contrast, Lithuania's closure of the Department left physical activity policy fragmented, with no clear governance structure or strategy in place to address growing public health concerns related to inactivity.

On the 1st of January 2019, a new version of the Law on Sport came into force: it established the principles of sport, the system of sport and its governance, regulated the competences of state and municipal institutions and bodies in the field of sport, the requirements for specialists in physical activity or high performance sport and for instructors in physical activity or high performance sport, the organisation of sport, the health check of persons, the requirements for safety of sport events, the financing of sport, the implementation of the policy of antidoping and the policy of fighting against the manipulation of high performance sport competitions, and the promotion of the State for the achievement of achievements in high performance sport. The Law on Sport provides that the state sport policy will be formulated, coordinated and implemented by the Ministry of Education and Science, which as of 1 January 2019 became the Ministry of Education, Science and Sport. However, the new version of the Law on Sports came into force from the 1st of January 2025.

In 2022, Lithuania established the National Sports Agency to implement national sport policy in high-performance sport and physical activity. However, overlapping responsibilities exist with the Ministry of Education, Science and Sport. The Agency primarily handles administrative functions—such as promoting and financing sport—but has limited authority to enforce the Law on Sport. According to the law, sports monitoring is carried out by the Ministry and/or its authorized institutions, municipal administrations, and sports federations (Article 5). Various state institutions, beyond the Ministry and the Seimas, also contribute to sports development based on legal mandates. The National Sports Agency is tasked with specific roles but does not shape national sports policy. This responsibility lies with the National Sports Council, a collegial body advising the Seimas, Government, and Ministry on strategic sports issues, including national strategy and priority areas (Article 7). However, the Council currently does not fulfil its legally mandated functions. Thus, while the Agency executes policy, the formulation of policy remains under the purview of the Council and the Ministry, highlighting gaps in coordination and implementation under the current governance structure.

As Begovic (38) highlights, a key shortcoming in Lithuania's sport policy is the lack of a long-term, systematic implementation approach. State institutions often fail to apply principles such as consistency, clear responsibility, public engagement, and inter-institutional cooperation. Sport policy lacks integration with scientific progress and community needs, limiting its effectiveness. To address this, cooperation models between public and private sectors and targeted action plans must be developed urgently. The Law on Sport designates sports organisations as key actors in promoting physical activity and healthy lifestyles, yet the normative framework focuses mainly on athlete safety and event organisation. Scientific potential remains underused, with inadequate funding for athlete development and no evidence-based monitoring or strategic planning in sport policy.

In summary, Lithuania's sport policy continues to face structural and regulatory barriers characterized by unstable institutional oversight, shifting ministerial responsibilities, unrealized governance reforms, and insufficient coordination mechanisms, all of which undermine policy continuity and effective implementation.

## Politicization and its impact on sport governance

Public investment in sport in Lithuania has increased in recent years, sparking debate over resource allocation. However, there is limited national analysis of the factors influencing sport policy or the variables shaping its development. The sport sector remains largely uncoordinated, with poor horizontal integration across public administration, municipalities, NGOs, and the private sector. Policy actions are mostly internal to the sport sector, disconnected from broader state strategic goals. For instance, physical education is tied only to formal schooling objectives, and sport development programs fail to reflect sport's wider social value or align with national priorities. Municipalities, which should play a key role in community sport development, often lack the capacity to implement the responsibilities outlined in the Law on Sport (Article 8). While the law empowers municipalities to set long-term sport goals and foster public-private partnerships based on national strategy and community needs, the absence of a clear national sports strategy and vague sport priorities (as seen in the Sports Development Program 2021–2030) hinder effective implementation. As a result, municipalities struggle to realize their potential in shaping and sustaining local sport policies and initiatives.

Although the role of private business is noted, especially when it comes to private investments in sports infrastructure, its importance for the development of the sports movement and cooperation with socially responsible business remains unrecognized and unrelated to public policy priorities that are not related to the development of sport policy [Program of the Government of the Republic of Lithuania (45) Lithuanian Progress Strategy “Lithuania 2030” (46) State Progress Strategy Lithuania's Future Vision “Lithuania 2050” (47)].

The independent role of clubs and NGOs in Lithuanian sport is undervalued. Although the Ministry of Social Security and Labor encourages cooperation between municipalities and NGOs in public service delivery, effective partnerships require institutionalized processes with NGO involvement from start to finish. Challenges include municipalities' dependence on unclear state sport policy guidelines and the limited capacity of NGOs, leading to isolated efforts. Meanwhile, key administrative functions remain with the Government and Ministry of Education, Science and Sports, which have yet to perform these roles effectively, hindering coordinated sport development (Lithuania's 2030 Progress Strategy).

The fight against corruption is one of the guarantees of democracy and the rule of law. As noted in the European Commission's report (2018), Lithuania has made significant progress in prosecution, but at the same time it has not made significant breakthroughs in the formulation of policies to prevent corruption, and there is a lack of effective practical implementation of the prevention of corruption in particular, which calls for a continuation of the work started to tackle the risks of political corruption (48).

International norms state that the exercise of a public function, e.g., representing public/local authorities or a political organisation, is incompatible with a role in a non-profit organisation or NGO (49), ETS 173; (50), CETS 191; (51), ETS 174). Article 14 of the Law on Associations of the Republic of Lithuania, on the basis of which sports federations operate, states that state and municipal authorities and officials in cases and procedures not prescribed by law, political parties and political organisations, other organisations and individuals are prohibited from interfering in the activities of an association and in its internal affairs (52). Lithuanian legislation does not allow representatives of political parties or public institutions to occupy key positions in the governance structures of sports organisations. This institutional framework does not prevent the active fight against corruption in and through sport, which has a positive impact on the governance of sport.

The National Sports Agency in Lithuania is carrying out competency development cycles related to the analysis of the activities of municipal sports training centres in Lithuania and the preparation of best practice recommendations for the activities of sports training centres in Lithuania, the implementation of the principles of good governance, the development of the employment relationship between sportsmen and women, and the preparation of strategic action plans. Sports federations are proactively working on their operational strategies as a prerequisite for receiving state budget funding for high-performance sport programs. While there is legislation designed to promote and facilitate the fair resolution of disputes in sport, none of the federations have implemented anonymous complaint or confidentiality procedures to ensure impartial treatment of all parties involved.

In 2018, the National Athletes' Association was set up due to a difficult situation in which athletes started to fear for their future and the main sports institutions did not always listen to athletes. One of its main objectives is to unite the representatives of the different sports, to defend their rights and represent the interests of athletes, to make the country's sports institutions transparent, and to work together to build the future of athletes. As of 2020, the European Athletes' Union will be responsible for the development of the European Athletes' Union. Founded in Europe in 2007, the National Athletes' Association (NAA) is also a member of EU Athletes, an association of more than 25,000 athletes, with the aim of uniting athletes from different sports and representing their interests. Sports clubs operate under the Public Bodies Act of the Republic of Lithuania. A public body is a non-profit-making public legal person with limited civil liability, the purpose of which is to serve the public interest by providing public services and/or carrying out other activities of benefit to the public (Law on Public Bodies, 1996).

## Implementation of internationally binding standards

Each area has international standards outlining the official procedures to be followed. Combating doping is crucial for safeguarding the health of athletes and sports professionals. Ratifying international doping standards requires the relevant national sports authority to establish a National Commission against Doping in Sport, which will be responsible for overseeing the enforcement of anti-doping regulations (39).

In 1989, Lithuania acceded to the Anti-Doping Convention (53) in order to reduce the number of doping athletes and to put a definitive end to doping, and undertook to take the necessary steps to implement the provisions of the Convention in its relevant constitutional resolutions (16).

In 1995, the Seimas of the Republic of Lithuania ratified the Council of Europe Anti-Doping Convention, thus declaring its position in favour of clean and fair sport. To reinforce this position, the Seimas of the Republic of Lithuania also ratified the Additional Protocol to the Council of Europe Anti-Doping Convention on 28 September 2004 (17).

On 10 July of the same year, the Government of the Republic of Lithuania signed the Copenhagen Declaration on Anti-Doping in Sport, by which Lithuania committed to implement the World Anti-Doping Agency Code. The Seimas of the Republic of Lithuania ratified the International Convention against Doping in Sport on 2 May 2006 (54). Confirmation of this has been sent to the World Anti-Doping Agency (WADA), to the Danish Minister of Culture, who is responsible for the accession of the European Union Member States to this declaration, and to the International Olympic Committee (55).

The Seimas of the Republic of Lithuania ratified the International Convention against Doping in Sport on 2 May 2006 (56).

In order to ensure the smooth running of the 27 July 2005 The Department of Physical Education and Sports under the Government of the Republic of Lithuania established the Public Institution “Lithuanian Anti-Doping Agency”. The vision of the institution is to establish a responsible anti-doping programme in the Republic of Lithuania, thereby achieving fairness and equality among athletes. The mission is to promote transparency in the sports competition environment by implementing programs aimed at preventing the use of banned substances. The Agency participates in events organised by the Council of Europe, the Council of Europe, UNESCO and WADA, in the framework of the implementation of the World Anti-Doping Code, the Council of Europe Anti-Doping Convention, the Copenhagen Declaration and the UNESCO International Convention against Doping in Sport. To strengthen its global position in the fight for clean sport, the Lithuanian Anti-Doping Agency has been a member of the Institute of National Anti-Doping Organisations (iNADO) since 2014 (57).

With the Lisbon Treaty, the EU gained a legal basis to act in sport through Article 6(e) Treaty on the Functioning of the European Union (TFEU) (58), designating it as a supporting competence. Article 165 TFEU outlines the Union's role in promoting European sporting issues, respecting sport's voluntary nature and social function. It emphasizes developing the European dimension in sport, ensuring fairness, openness, and athlete protection, especially for youth. Article 165(3) promotes cooperation with third countries and international bodies like the Council of Europe. These provisions support initiatives like Erasmus + Sport, EU sport diplomacy, and coordination through the Education, Youth, Culture and Sport Council (EYCS), while linking sport to broader policy goals in health, education, and social inclusion.

In 2021, the International Standard List of Prohibited Substances and Methods of the World Anti-Doping Code was adopted by the LNOC as part of the World Anti-Doping Programme (59).

The Law on Sport defines what an Anti-Doping Organisation is, as defined on 19 October 2005. The International Convention against Doping in Sport, ratified by the Law of the Republic of Lithuania No X-591 of 2 May 2006 “On the ratification of the International Convention against Doping in Sport”. Furthermore, the Law defines the objective of the Anti-Doping Programme as a four-calendar-year planning document which sets out the activities of the National Anti-Doping Organisation in relation to the implementation of the World Anti-Doping Code, the measures to implement them, the timeframe for the implementation of the measures, the need for funds for the implementation of this document in each of the current calendar years, and the planned sources of these funds (33).

26 November 2019 The Rules of Procedure of the Arbitration for Sport under the SCAT (SA Rules) were approved in Lithuania. The SA Regulation has the peculiarity that it regulates the appeal procedure for the final resolution of a dispute when an arbitration clause is included in the statutes or other operational regulation, document and/or competition rules of a sports organisation or its body. It is expected that the new SA Regulation will be more in line with the need of Lithuanian and foreign entities to resolve disputes arising in the field of sport (60).

On the 1st of April in 1993, the Republic of Lithuania signed and, by Law No VIII-1625 of 13 April 2000, ratified the 1985 European Convention on the Brutal Treatment of Spectators at Sports Competitions, and in Particular at Football Matches, which focuses on the prevention of, deterrence of, and response to, violence and ill-treatment in stadiums and their vicinity. One of the key elements of the Convention in combating all forms of violence and discrimination is increased cooperation between the various stakeholders (61).

The Law on Sport (Articles 14 and 15) sets clear requirements for organizing and conducting sporting events, focusing on security and safety. Event security rules must address conditions for spectators and participants entering and leaving the venue, spectator zones, restricted areas, evacuation routes, and safe conduct during the event. They must also include specific measures to maintain order and safety in and around the venue, and details on the presence and location of medical and fire-fighting services if involved. Organizers must approve the event regulations and safety rules and obtain approval from the municipal authority responsible for sporting events, as designated by the municipal council. For higher-risk events, organizers must notify the relevant territorial police authority in writing at least 20 working days before the event and submit the security rules. The Minister of the Interior establishes the criteria for defining a higher-risk sporting event. These regulations aim to ensure the safety and smooth conduct of sporting events while coordinating responsibilities among organizers, municipalities, and law enforcement. Thus, general standards and safety measures are provided for in the European Convention on the Brutal Treatment of Spectators at Sporting Events, and in particular at Football Matches (62), the Law on Meetings of the Republic of Lithuania (63), and the Law on Sport (64).

However, these laws do not include a specific licensing procedure for safety protocols for stadiums for events. Therefore, the National Sports Agency determines each year the priorities and specific evaluation criteria for the funding of national and regional physical activity projects and projects for the improvement of sports facilities from the state budget (44). In this context, Lithuania has a clear division of jurisdiction/competence between state and local government sport authorities (Council of Europe, 1985, CETS 120).

As shown in 2021 performed in 2011–2020 National sports development 2011–2020 review of strategy implementation, sports development was not linked to the systematic renewal and construction of sports infrastructure (both geographically and according to the specialized needs of the developed sports). The regional and municipal level of sports development was partially ignored. During that period, the state invested insufficiently in the international competitiveness of the country's sports, as far as the physical conditions for training high-quality athletes were concerned. Lithuanian athletes were not given full opportunities to compete with athletes from foreign countries (this increased the probability of emigration of talented Lithuanian athletes) (Overview of the implementation of the National Sports Development Strategy 2011–2020, 2021).

On the 9th of September 2020, the Government approved the 2021–2030 the national progress plan, which aims to determine the main changes aimed at in the next decade in the country, ensuring progress in the social, economic, environmental and security areas, and to mobilize funding sources for the implementation of these changes.

The plan envisages long-term strategic goals, progress objectives and indicators for measuring quantitative progress with target values for the year 2030.

The links between the 2021–2030 National Progress Plan Lithuanian Progress Strategy “Lithuania 2030” (46) and the goals of sustainable development are very important. One of the strategic goals is to increase the social well-being and inclusion of the population, strengthen health and improve the demographic situation of Lithuania. reduction of poverty and social exclusion, general health indicators are analyzed. i.e., to improve public health—to develop a responsible attitude to health, to increase the physical activity of children and adults, as this reduces the prevalence of bad habits and determines the health status and length of life, but there are no indicators of the impact of the second strategic goal realized.

In 2024, under Article 21(3) of Lithuania's Sports Law, the Minister of Education, Science and Sports approved regulatory recommendations for sports training institutions funded by municipal budgets, aiming to ensure good management and improve performance. While this is a key step in sports policy development, it is insufficient. The social role of sports remains undervalued, with emphasis placed mainly on sports achievements and their contribution to the country's international image and national identity. Although important, this focus overlooks sports' broader social impact on community and societal development.

## Conclusions

This study aimed to examine how Lithuanian sport policy has evolved since regaining independence and to identify the political and institutional factors shaping its development. The findings reveal that while structural reforms have taken place, the sport system remains heavily centralized, politically influenced, and lacking in long-term strategic vision. Sport, and its development, is not recognised as a key priority of the state, as a distinct branch of the economy, designed to promote healthy lifestyles, improve the quality of life, health and social cohesion. The absence of a comprehensive national sport strategy, overlapping institutional responsibilities, and limited stakeholder engagement—particularly the exclusion of athletes, coaches, and civil society—have hindered effective governance. Political instability and frequent administrative restructuring have also disrupted policy continuity. Placed in broader regional context, challenges are echoes of what has been seen elsewhere in the post-socialist world. Both Latvia and Estonia had trouble developing longer-term sport strategies early on in independence, but Estonia was quicker to stabilize funding and coordination, whereas Lithuania continued to struggle with shattered governance. The same issues are present all over Central and Eastern Europe, as the sport has often been left behind in economic and social reform. But serial failure to enact a national policy places Lithuania near the weaker end of the scale, pointing to its low capability for elevating sport policy to the level of being a bona fide state priority. To address these issues, the study recommends the development of a unified, long-term national sport policy aligned with health, education, and inclusion objectives; enhanced multi-level coordination across government, municipal, and non-governmental actors; institutionalized stakeholder involvement in decision-making processes; performance-based and transparent funding mechanisms; and the integration of scientific evidence in policy planning and evaluation. Given this context, it is improbable that the current bureaucratic leadership, predominantly composed of political figures, will possess either the motivation or the legitimacy to initiate a dialogue that could lead to the necessary structural changes. These actions are essential for building a more inclusive, accountable, and future-oriented sport governance system in Lithuania.

## Data Availability

The original contributions presented in the study are included in the article/Supplementary Material, further inquiries can be directed to the corresponding authors.
